# Investigation of the Relationship between Psychological Variables and Sleep Quality in Students of Medical Sciences

**DOI:** 10.1155/2017/7143547

**Published:** 2017-09-28

**Authors:** Majid Najafi Kalyani, Nahid Jamshidi, Javad Salami, Elahe Pourjam

**Affiliations:** ^1^Department of Medical Surgical Nursing, School of Nursing and Midwifery, Shiraz University of Medical Sciences, Shiraz, Iran; ^2^Student Research Committee, Faculty of Nursing & Midwifery, Shiraz University of Medical Sciences, Shiraz, Iran; ^3^Lamerd Nursing School, Shiraz University of Medical Sciences, Shiraz, Iran

## Abstract

**Objectives:**

Students of medical sciences are exposed to many emotional and mental problems. In light of the importance of sleep quality in learning and liveliness, this study was conducted to examine the relationship between psychological variables (stress, anxiety, and depression) and sleep quality of students.

**Design:**

This research is a cross-sectional analytical study, where all students studying at Fasa University of Medical Sciences in 2012-2013 year were selected. To examine the students' stress, anxiety, and depression values, the standardized 21-item DASS-21 was used, and to examine their sleep quality, Pittsburgh Sleep Quality Index* (PSQI)* was used.

**Results:**

The results of the study demonstrated that 73% of the students have moderate and severe stress, and 46.4% of them have PSQ scores ≥ 5. The students' mean sleep quality score was 4.65 ± 2.37, and their stress score was 8.09 ± 5.14. A statistically significant relationship was found between the students' stress levels and sleep quality (*P* < 0.001).

**Conclusion:**

The high stress levels decrease students' sleep quality. High stress levels and also the significant relationship between stress value and decrease in students' sleep quality call for more attention to and care for students' emotional and mental issues and timely proper interference on the part of authorities.

## 1. Introduction

As a young population, university students are the major assets of every country [1]. It is a critical period in a youngster's life when he enters the university, often accompanied by several changes in their social and interpersonal relations [2]. Many students will be responsible in the future for education of generations after them, and thus, they also indirectly interfere with education of later generations of the society [3]. A society concerned by its health and its future generations' should take steps more than ever toward achievement of ideals by examining and removing factors allowing and making emotional and mental problems or preserving the health of makers of its future [3]. In light of the fact that the country's university student population constitutes a considerable part of the population and that it is increasing every year, the significance of university students' health becomes clearer than ever [4]. Here, it is crucial to pay attention to students of medical science fields, who will be responsible for the society's health in the future [3]. Mental health is an important dimension of health, provision of students with which causes their progress and success [3, 4]. Due to their age conditions and particular social position, students are exposed to numerous stresses. Factors like being away from the home environment, separation from family, entering a new environment, educational issues and problems, competition with other students, working future, and dormitory life can be considered as stresses for students [3]. Chronic, long-term stress can lead to occurrence of physical and mental diseases [3]. People with high stress levels suffer in their lives from degrees of other psychological problems like anxiety and depression. Besides, problems they make for students during education as mental health factors, stress, anxiety, and depression cause interference with professional roles and undertaking responsibility for health of people in the society in future. Therefore, prevention of students' stress, anxiety, and depression and reduction of mental pressure will play an important role in enhancement of interest in work and cooperation in groups and feeling of responsibility [4, 5].

Sleep is one of anyone's physiological and fundamental needs, quality of which is related to health [6]. Quality and quantity of sleep can easily be altered in people with changes in physical and social conditions [7]. Night sleep disorder and consequently a fall in sleep quality cause occurrence of problems such as drowsiness and boredom during the day, stress and anxiety, headaches, and also weak performance in educational and academic plans. Insufficient sleep and sleep deprivation cause neurological, behavioral, and physiological changes, occurrence of academic failure, and decrease in normal routine performance in class, and they consequently prevent students from participation in class and cause them to be drowsy during class participation [8]. Therefore, sleep disorder causes multiple scientific, behavioral, and emotional damage to occur in students [9]. In light of effect of sleep quality on performance, students' academic conditions are expected to be enhanced as well with improvement of their sleep conditions [10]. People with sleep disorder suffer from problems such as fatigue, cellular repair difficulty, memory and learning impairment, increase in tension and anxiety, and decrease in routine life quality. Moreover, low sleep quality increases depression and anxiety suffering and decreases the ability to confront routine tension [11–13].

Different pieces of research have demonstrated that high levels of stress, anxiety, and depression can leave negative effects on students' health, life quality, sleep quality, academic progress, and also readiness for undertaking their professional roles, and it is of particular importance to pay attention to it and its consequences as well as adopt proper solutions to get rid of it [4, 5]. In view of the importance of sleep quality in students of medical sciences and that stress, anxiety, and depression cause problems to occur in their personal and scientific lives, this study was conducted to examine the relationship between students' stress, anxiety, and depression values and their sleep quality.

## 2. Methods

This research is a descriptive analytic study, conducted within 2012-2013 academic year. The samples of this research include all students studying at Fasa University of Medical Sciences in 2012-2013 academic year for the three degrees Doctorate, bachelor's, and associate. The sampling was performed in the form of a census from all students after obtaining written consent of students and accurately clarifying the purpose of the study to them. The data collection instrument in this research included three sections: Demographic Information Questionnaire, the questionnaire concerning depression, anxiety, and stress assessment (DASS-21) (Depression, Anxiety, Stress, Scale), and Pittsburgh Sleep Quality Index* (PSQI)* questionnaire. Demographic Information Questionnaire contained information such as age, gender, field of study, academic degree, marital status, and interest in the field of study. To assess students' stress, anxiety, and depression, DASS-21 standardized questionnaire was used. The questionnaire was first presented by S. H. Lovibond and P. F. Lovibond in 1995 [14], containing 21 Likert-Scale questions, 7 questions concerning stress, 7 concerning anxiety, and 7 concerning depression. In Iran, the reliability of the instrument in a sample of the public population of the city Mashhad (400 people) has been reported as 0.70 for the depression value, 0.66 for anxiety, and 0.76 for stress [15]. The validity and reliability of the instrument have been tested and confirmed in Iran in different studies by Aghebati [16], Moradipanah [17], Jamshidi et al. [18], and Rezaei-Adryani et al. [3]. S. H. Lovibond and P. F. Lovibond (1995) have also suggested the high correlation of the questionnaire with similar ones [14]. In this research, the scale's internal consistency value was specified using Cronbach's alpha, which was 0.87 for the stress value, 0.84 for anxiety, and 0.82 for depression. To assess sleep quality, Pittsburgh standardized questionnaire was used, the validity and reliability of which have been confirmed in studies inside and outside Iran [6, 7]. The questionnaire examines people's sleep quality in the past 4 weeks. In the questionnaire, seven scales with scores 0 to 3 are assessed and obtain a total score between 0 and 20. A total score of 0 to 4 was considered as desirable sleep quality and a score of 5 to 21 as undesirable sleep quality [7, 11]. After the data collection, the data analysis was conducted by entering the information into SPSS software version 15 and using tests of descriptive statistics and inferential statistics such as *t*-test, chi-square, One-Way Analysis of Variance, and the correlation coefficient. An alpha level of 0.05 was considered as the significance level.

## 3. Results

After distributing questionnaires to all studying students, 278 students filled out the questionnaires and returned them. Among the students participating in the research, 97 (35%) were male, and 181 (65%) were female. The mean age was 19.98 ± 1.83 years for the male students and 19.83 ± 1.34 for the female ones. From the aspect of marital status, 239 (86%) were single, and 39 (14%) were married. From the aspect of academic degree, 195 (70.1%) were bachelor's, 53 (19.1%) were Doctorate, and 30 (10.8%) were associate degree students. From the aspect of field of study, the highest number of participants in the study was nursing (61, 21.9%) and the lowest number was medical emergency (29, 10.4%) students. 252 (90.6) were dormitory residents, and 25 (9%) were nondormitory residents. 12 (4.3%) of students mentioned positive smoking history. From the aspect of interest in the field of study, 122 (43.9%) mentioned relative interest in their fields of study, 112 (40.3%) full interest, 33 (11.9%) relative disinterest, and 11 (4%) full disinterest.

The results obtained from the study demonstrated that 72.3% of students have stress (47.5% moderate stress and 24.8% severe stress), 40.3% have anxiety (32.4% moderate anxiety and 7.9% severe anxiety), and 51% have depression (36.3% moderate depression and 14.7% severe depression). From the aspect of sleep quality, 53.6% of students had PSQ scores ≤ 4, and 46.4% had PSQ scores ≥ 5 (Table 1).

The data analysis using independent *t*-test demonstrated that the mean stress and depression values are lower in male students than in female ones, but the difference is not statistically significant (*P* > 0.05). The mean anxiety is lower in male students than in female ones, and the difference is statistically significant (*P* > 0.01). The mean sleep quality is also lower in male students than in female ones, but the difference is not statistically significant (*P* > 0.05) (Table 2).

As for comparison of students' stress, anxiety, and depression scores in view of their academic degrees and based on ANOVA statistical test, the results obtained from the study demonstrated that the mean stress and anxiety are not statistically significant between the three degrees (*P* > 0.05), but from the aspect of depression value, there is statistically significant difference between the three degrees associate, bachelor's, and Doctorate (*P* < 0.05). Moreover, by examining sleep quality of students for the three degrees, it was found that there is statistically significant difference between the three groups (*P* < 0.05) (Table 3).

As for investigation of the relationship between the variables under study based on Pearson's correlation coefficient, it was found that students' stress, anxiety, and depression are statistically significantly related to sleep quality (*P* < 0.05). Thus, students' mean sleep quality scores also increase with increase in their mean stress, anxiety, and depression, which demonstrates their PSQ scores ≥ 5. A statistically significant relationship was also found between the three psychological variables in the study, which demonstrates the direct relationship between these variables (Table 4).

The results of this study showed that the stress, anxiety, and education levels of students affect the quality of sleep after adjusting, but sex and depression did not show a relationship with sleep quality (Table 5).

## 4. Discussion

The results obtained from the study demonstrated that 72.3% of students suffer stress (moderate and severe), and 40.3% suffer anxiety (moderate to severe). The study performed by Rezaei-Adryani and colleagues [3] on students residing in student dormitories demonstrated that 71.7% suffer stress, and 39.5% suffer anxiety. Studies conducted in the area demonstrate that students suffer high levels of stress and anxiety [3, 4, 8, 12]. The results obtained from the present study demonstrate that students suffer psychological problems related to the education period and life as students. Slight differences between stress and anxiety values in different studies can be attributed to environments where research has been conducted and to support resources and facilities of universities.

It was found in the study that 51% of students of medical sciences suffer depression (moderate to severe). Rezaei-Adryani and colleagues demonstrated in their study that the depression value in students residing in dormitories of medical sciences is 51.6% [3]. Furthermore, the study conducted by Furr and colleagues has also reported that 53% of students suffer depression [19]. The results obtained from the present study in the area of students' depression are in accordance with those of other research conducted in the area.

The results obtained from this study demonstrated that female students' mean stress, anxiety, and depression values are higher than those of male students. Studies conducted by Grant [20] and Watanabe [21] demonstrate that scores of psychological variables are higher in females than in male students. These results are in accordance with that obtained in the present study. Contrary to the result obtained in this study, stress, anxiety, and depression levels are higher in male students in Rezaei-Adryani and colleagues' study [3]. The difference can be attributed to the field type, cultural environment and society, support facilities, and other underlying causes. Studies conducted in the area demonstrate that increase in personal, educational, economic, social, and cultural stressors causes students' anxiety and depression values to increase. Moreover, high stress, anxiety, and depression levels in students will cause performance decrease and academic failure in them [3, 5].

Through investigation of the study variables in students' different academic degrees, it was found that mean stress, anxiety, and depression values are lower in Doctorate degree students than in bachelor's degree ones. These results are different from those obtained in Rezaei-Adryani et al.'s study [3]. They found in this study that there is no significant difference between the two groups of master's and Doctorate students from the aspect of stress, anxiety, and depression values [3]. The lower values in Doctorate degree students can be attributed to their academic conditions and satisfaction with current conditions.

The results obtained from this study demonstrated that nearly half of the students (46.4%) have PSQ scores ≥ 5. Lima and colleagues [22] have reported in their study of sleep patterns of students of medicine in Brazil that 42.3 percent of students are confronted with sleep disorder. In their study on Brazilian students, Mesquita and Reimão [23] reported sleep quality as undesirable in 60.38 percent of students and as desirable only in 39.72 percent. In a study performed on students of medicine, Ardani Rezaie and colleagues [24] also demonstrated that 39.8 percent of students are faced with PSQ scores ≥ 5. In Nojoomi and colleagues' [25] study on specialized assistants, interns, apprentices, and students of basic sciences and physiopathology, it was demonstrated that among sleep disorders, insomnia, and among types of insomnia, interrupted sleep (49 percent) has the highest incidence in students of different categories of medicine. And among types of parasomnia, some had suffered nightmare (32 percent), sleep eating (1 percent), somnambulism (1.3 percent), somniloquy (7.5 percent), and bruxism (10.2 percent). Research performed by Farhadinasab and Azimi [26] on students of medicine demonstrated that 48 percent of students are confronted with sleep disorder. Also, in another study performed by Ghoreishi and Aghajani [27] on students of medicine, it was found that 40.6 percent of students of medicine are confronted with PSQ scores ≥ 5. The results of the above research are in accordance with those obtained from the present study and demonstrate that students of medical sciences have PSQ scores ≥ 5.

The results obtained from the present study demonstrated that there is a statistically significant relationship between students' stress, anxiety, and depression values and their sleep quality, such that their sleep quality decreases as stress, anxiety, and depression values increase. Vandeputte and de Weerd [28] have demonstrated in their study that feeling of depression is accompanied by sleep disorder. It has also been found in some other studies that students sleep less than the general public, which may be due to their higher stress and concern or due to educational pressure and for more study [29]. In Lowry and colleagues' research [30] on students of University of Minnesota, a significant relationship has been reported between students' mean night sleep and mean scores, such that students' mean scores have shown increase as sleep hours have increased. Another study suggests that weak sleep quality is directly related to students' behavioral and physical performance and that enhancement of sleep hygiene can help remove students' sleep problems [31]. New studies have found that insomnia is also a risk factor for depression to progress, decreases response to treatment of depression, and increases recurrence probability of depression [28]. In a study conducted by Doi and colleagues, it was demonstrated that sleep quality is at a lower level in people with depression and anxiety history, but the difference is not statistically significant [32]. Therefore, results of the studies mentioned are in accordance with that of the present study, and this demonstrates that psychological problems in students decrease their sleep quality.

One of the limitations of this study can be stated as use of self-report method for evaluation of students' psychological variables. It is suggested that, in later studies, interview and clinical examination also be used besides self-report tools for diagnosis of students' psychological problems. Another limitation of the study was the small sample size since few questionnaires were returned by students. It is suggested that, in future studies, larger samples be used for better conclusion and generalization.

## 5. Conclusion

In view of the findings obtained from the study, it can be concluded that there are many psychological problems in students and that particular planning and attention are required on the part of authorities. Students' undesirable sleep quality and its relationship with increase in stress, anxiety, and depression values cause these students, who should be responsible for the society's health in the future, to have problems at the university and work. Authorities and those in charge can take steps toward enhancement of students' emotional and mental health by identifying students' problems and removing them.

## Figures and Tables

**Table 1:** Prevalence of stress, anxiety, and depression based on gender.

Variable		Stress			Anxiety			Depression			Sleep quality	
		Normal	Moderate	Severe	Normal	Moderate	Severe	Normal	Moderate	Severe	Desirable	Undesirable
Male	%	34.0	45.4	20.6	68.0	29.9	2.1	51.5	37.1	11.3	61.9	38.1
Female	%	24.3	48.6	27.1	55.2	33.7	11.0	47.5	35.9	16.6	49.2	50.8
Total	%	27.7	47.5	24.8	59.7	32.4	7.9	48.9	36.3	14.7	53.6	46.4

**Table 2:** Comparison of the mean scores of stress, anxiety, depression, and sleep quality of students.

Variable	Female	Male	*P* value
	Mean ± SD	Mean ± SD	
Stress	8.57 ± 5.15	7.20 ± 5.03	0.894
Anxiety	5.28 ± 4.39	3.52 ± 3.40	0.001
Depression	6.29 ± 5.39	5.20 ± 4.84	0.269
Sleep quality	4.87 ± 2.39	4.24 ± 2.28	0.140

**Table 3:** Comparison of the mean scores of stress, anxiety, depression, and sleep quality among students based on their studying grade.

Variable	Degree			*P* value
	Associate degree	Bachelor's degree	Doctorate degree	
	Mean ± SD	Mean ± SD	Mean ±SD	
Stress	6.23 ± 5.05	8.51 ± 5.08	7.62 ± 5.21	0.059
Anxiety	3.90 ± 3.31	4.89 ± 4.29	4.25 ± 4.05	0.344
Depression	4.17 ± 4.25	6.43 ± 5.38	4.96 ± 4.84	0.029
Sleep quality	4.37 ± 2.14	4.89 ± 2.46	3.94 ± 2.02	0.028

**Table 4:** Results of partial correlation between sleep quality, stress, anxiety, and depression.

	Sleep quality	Stress	Anxiety	Depression
Sleep quality	1			
Stress	0.206	1		
Anxiety	0.198	0.375	1	
Depression	0.116	0.657	0.436	1

**Table 5:** Results of linear regression of effects of stress, anxiety, depression, sex, and education level on sleep quality.

	*β*	St. Err *β*	*P* value
Stress	0.041	0.027	0.034
Anxiety	0.064	0.032	0.017
Depression	0.022	0.026	0.105
Sex	0.039	0.034	0.298
Education level	0.058	0.028	0.003
